# Nighttime Appendectomy is Safe and has Similar Outcomes as Daytime Appendectomy: A Study of 1198 Appendectomies

**DOI:** 10.1177/1457496920938605

**Published:** 2020-07-14

**Authors:** T. Mönttinen, H. Kangaspunta, J. Laukkarinen, M. Ukkonen

**Affiliations:** 1 New Children’s Hospital, Helsinki, Finland; 2 Faculty of Medicine and Health Technology, Tampere University, Tampere, Finland; 3 Department of Gastroenterology and Alimentary Tract Surgery, Tampere University Hospital, Tampere, Finland

**Keywords:** Appendectomy, appendicitis, surgery, morbidity, mortality, efficiency, organizational

## Abstract

**Introduction:**

Although it is controversial whether appendectomy can be safely delayed, it is often unnecessary to postpone operation as a shorter delay may increase patient comfort, enables quicker recovery, and decreases costs. In this study, we sought to study whether the time of day influences the outcomes among patients operated on for acute appendicitis.

**Materials and Methods:**

Consecutive patients undergoing appendectomy at Tampere University Hospital between 1 September 2014 and 30 April 2017 for acute appendicitis were included. Primary outcome measures were postoperative morbidity, mortality, length of hospital stay, and amount of intraoperative bleeding. Appendectomies were divided into daytime and nighttime operations.

**Results:**

A total of 1198 patients underwent appendectomy, of which 65% were operated during daytime and 35% during nighttime. Patient and disease-related characteristics were similar in both groups. The overall morbidity and mortality rates were 4.8% and 0.2%, respectively. No time categories were associated with risk of complications or complication severity. Neither was there difference in operation time and clinically significant difference in intraoperative bleeding. Patients undergoing surgery during night hours had a shorter hospital stay. In multivariate analysis, only complicated appendicitis was associated with worse outcomes.

**Discussion:**

We have shown that nighttime appendectomy is associated with similar outcomes than daytime appendectomy. Subsequently, appendectomy should be planned for the next available slot, minimizing delay whenever possible.

## Introduction

Acute appendicitis is the most common emergency gastrointestinal operation (1–3). While it is debated, surgical care is still the golden standard care for acute appendicitis (4). Acute appendicitis can be divided to uncomplicated and complicated disease. When there is an abscess or appendix perforation involved with the appendicitis, the disease is considered complicated. Complicated appendicitis is associated with higher morbidity, mortality, and longer hospital stay (5). According to the studies, 16%–38% of the acute appendicitis patients develop a perforation during the 36 h after their symptoms began and the risk remained at 5% for each following 12-h period (6).

In a recent meta-analysis (7) there was no significant difference in the 12- to 24-h delay in operation in terms of the acute appendicitis developing complication. However, the assumption is that the sooner the patients would get operated, the sooner they would also get home (6). Early surgery would reduce the costs of the treatment, especially from the aspect of the ward stay. As patients with acute appendicitis present to emergency department any time of day, minimizing the delay would sometimes mean that patients are required to be operated during nighttime. However, after normal working hours, fatigue and sleep deprivation may increase the risk of adverse outcomes (8). There are several reports from different surgical fields reporting worse outcomes for surgeries performed during after-office hours or nighttime (9). However, patient-related demographics among those operated during nighttime are often different than among those operated during daytime. These differences may explain some of the worse results.

In this study, we sought to study if nighttime surgery is associated with worse outcomes.

## Materials and Methods

In this retrospective study, consecutive patients operated at Tampere University Hospital between 1 September 2014 and 30 April 2017 for acute appendicitis were included. Patients were identified from the institutional database by retrieving all surgeries associated with the Nordic Medico-Statistical Committee (NOMESCO) classification of surgical procedures (Version 1.13) code “JEA00” (appendectomy) and “JEA01” (laparoscopic appendectomy). Hospital records including surgical and histopathological reports were carefully retrieved from each patient individually. Patients with negative appendectomies were excluded. Appendicitis was considered complicated if there was perforation either with peritonitis or abscess.

## Study Hospital

In-house emergency surgeons working in a high-volume tertiary care emergency center with the hospital catchment area exceeding 1 million inhabitants operated all patients. All surgeons were experienced on acute care surgery and were either experienced residents or consultant surgeons. There was also a consultant surgeon available on-call at all times. In-house surgeons in the study hospital work in approximately 24-h shifts (7:30 a.m. to 8:00 a.m. during weekdays and 9:00 a.m. to 9:00 a.m. during weekends). In the study hospital, there was one operation theater available for the acute abdominal surgeries all the time during the study.

## Outcome

Patients were divided into two groups based on the time of day of the surgery. We used the time of day (beginning of the surgery) from 8 a.m. to 10 p.m. to define a daytime operation (Group A) and 10 p.m. to 8 a.m. to define a nighttime operation (Group B). Primary outcome variables were the following: postoperative morbidity, mortality (⩽30 days), operation times (minutes), amount of intraoperative bleeding (milliliters), and length of hospital stay (hours). Morbidity was defined and classified by using Clavien–Dindo (C-D) classification (10). Postoperative outcomes, including readmissions and complications, were accurately recorded from all regional health care facilities. The follow-up data were available for all patients.

## Ethical Aspects

The study was performed according to the Helsinki Declaration, and institutional review board approval was obtained.

## Statistical Analysis

All statistical analyses were performed using SPSS Statistics version 22 for Windows (IBM Corp, Armonk, NY, USA). Two-tailed chi-square tests were performed to compare categorical variables and Mann–Whitney U test for continuous variables. Multivariate analysis (binary logistic regression analysis, Enter-method) was used to identify risk factors associated with worse outcomes. Statistical significance was set at a p value less than 0.05.

## Results

A total of 1198 patients were included to this study, of which 65% (n = 776) were operated in daytime surgery group and 35% (n = 422) in nighttime surgery group. The overall morbidity and mortality rates were 4.8% and 0.2%, respectively. Patient- and disease-related demographics were similar in both groups, as shown in Table 1. Median time from treatment decision to surgery was 10 h (interquartile range (IQR): 4–18) in daytime surgery group and 5 h (IQR: 3–10) in nighttime surgery group (p < 0.001). When patients undergoing surgery in these two groups were compared, overall morbidity (4.8% vs 4.7%, p = 0.982) and 30-day mortality rates (0.1% vs 0.2%, p = 0.662) were similar. The length of hospital stay was shorter among those operated in nighttime surgery group (median, 40 h (IQR: 27–60) vs 37 h (IQR: 20–61), p < 0.001). None of the deaths were acute appendicitis or surgery-related.

**Table 1:** Patient and operation-related characteristics of the study population.

Variable	Nighttime surgery	Day-time surgery
	n = 422 (35%)	n = 776 (65%)
Age, median (min–max)	33 years (2–93)	34 years (2–91)
0–17 years	67 (16%)	93 (12%)
18–29 years	101 (24%)	213 (27%)
30–64 years	206 (49%)	391 (50%)
65–79 years	39 (9.2%)	65 (8.4%)
⩾80 years	9 (2.1%)	14 (1.8%)
Sex, female	185 (44%)	373 (48%)
ASA score I–II	376 (89%)	693 (89%)
ASA score ⩾III	56 (11%)	83 (11%)
CRP (mg/L), median (IQR)	37.1 (14.3–114)	46.1 (18.1–95.9)
WBC (109), median (IQR)	12.8 (10.0–15.7)	12.4 (9.9–15.7)
Delay before surgery, median (IQR)	5 h (3–10)	10 h (4–18)**
Laparoscopic appendectomy	345 (82%)	672 (87%)
Open appendectomy	69 (16%)	92 (12%)
Conversion from laparoscopic to open surgery	8 (1.9%)	12 (1.5%)
Uncomplicated appendicitis	316 (75%)	577 (74%)
Complicated appendicitis^a^	106 (25%)	199 (26%)
Abscess	17 (4.0%)	38 (4.9%)
Length of hospital stay, median (IQR)	37 h (20–61)	40 h (27–60)*
Morbidity	20 (4.7%)	37 (4.8%)
30-day mortality	1 (0.2%)	1 (0.1%)

ASA: American Society of Anesthesiologists; CRP: C-reactive protein; IQR: interquartile range; WBC: white blood cell.

a Complicated appendicitis = appendiceal perforation with either perforation or abscess.

* p value: 0.05–0.001; **p value < 0.001.

**Fig. 1.**
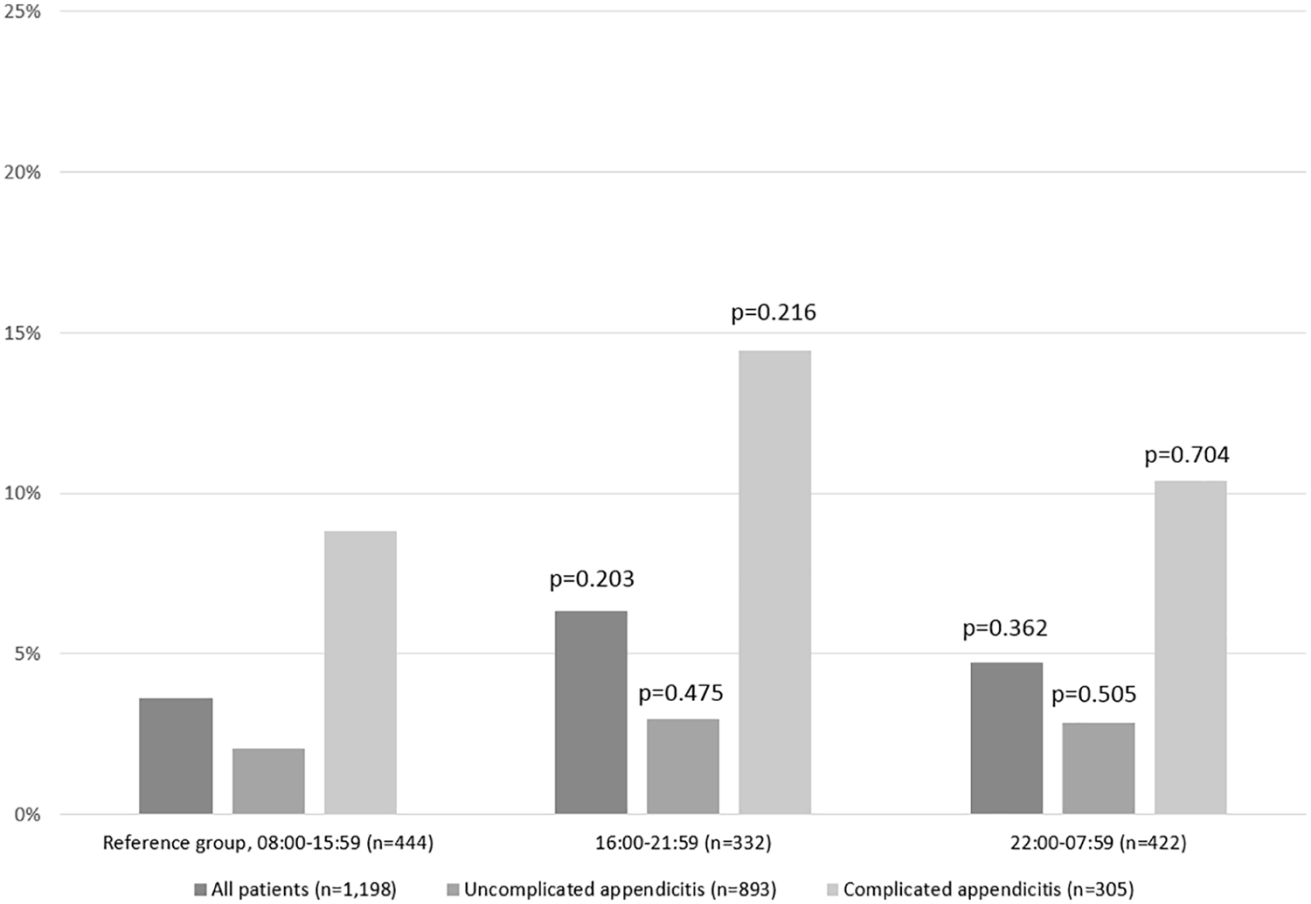
Postoperative morbidity among patients with uncomplicated and complicated acute appendicitis according to the time of day. Statistical comparison (chi-square test) between patients undergoing surgery during daytime (reference group) and nighttime (16:00–21:59 and 22:00-07:59).

Postoperative outcomes among those with complicated and uncomplicated acute appendicitis at different time of day are shown in Fig. 1. The most common postoperative complication was organ/space surgical site infection. Overall, the risk of these infections was similar in both groups (2.6% vs 2.6%, p = 0.976). Sixty-one percent of patients with postoperative abscesses required percutaneous drainage, 35% conservative care alone, and 3% underwent laparoscopic drainage. The median amount of intraoperative bleeding was less than 5 mL (range: 0–510 mL). While the amount of intraoperative bleeding was slightly higher among those undergoing daytime surgery, the risk of major bleeding that required blood transfusion was similar in both groups (0.2% vs 0.3%, p = 0.945). Neither there was association with other typical complications, including postoperative ileus (0.9% vs 0.6%, p = 0.728), pneumonia (0.4% vs 0.5%, p = 0.823), and superficial surgical site infections (0.6% vs 0.2%, p = 0.340). Severe complications (C-D III or more) were registered on 1.7% and 2.1% of patients (p = 0.573) and all these were related to organ/space surgical site infections requiring either percutaneous drainage or new laparoscopy.

A total of 199 patients (26%) in daytime surgery group and 106 patients (25%) in nighttime surgery group had complicated acute appendicitis. The overall morbidity among patients with complicated (12% vs 10%, p = 0.755) and uncomplicated acute appendicitis (2.4% vs 2.8%, p = 0.704) was similar in both groups, as shown in Table 2. The median length of hospital stay was shorter among those with uncomplicated appendicitis operated during nighttime (35 h (IQR: 23–43) vs 23 h (17–39), p < 0.001), while there was no statistically significant difference in the length of hospital stay among those with complicated disease.

**Table 2:** Postoperative outcomes among patients undergoing appendectomy.

Variable	Nighttime surgery	Daytime surgery	p-value
All AA patients	422 (35%)	776 (65%)	
Operation time, median (IQR)	40 min (28–55)	39 min (30–54)	0.140
Morbidity	20 (4.7%)	37 (4.8%)	0.982
Superficial SSI	1 (0.2%)	5 (0.6%)	0.340
Organ/space SSI	11 (2.6%)	20 (2.6%)	0.976
Ileus	3 (0.7%)	7 (0.9%)	0.728
Bleeding^a^	1 (0.2%)	2 (0.3%)	0.945
Pneumonia	2 (0.5%)	3 (0.4%)	0.823
Other complication	3 (0.7%)	2 (0.3%)	0.245
Clavien–Dindo I–II	11 (2.6%)	25 (3.2%)	0.355
Clavien–Dindo III–IV	9 (2.1%)	13 (1.7%)	0.573
30-day mortality	1 (0.2%)	1 (0.1%)	0.662
Length of hospital stay, median (IQR)	33 h (18–63)	37 h (25–63)	< 0.001
Complicated AA	106 (35%)	199 (65%)	
Operation time, median (IQR)	55 min (39–74)	54 min (35–65)	0.393
Morbidity	11 (10%)	23 (12%)	0.755
Superficial SSI	0 (0.0%)	1 (0.5%)	0.465
Organ/space SSI	8 (7.5%)	15 (7.5%)	0.998
Ileus	1 (0.9%)	6 (3.0%)	0.250
Bleeding^a^	1 (0.9%)	0 (0.0%)	0.170
Pneumonia	1 (0.9%)	1 (0.5%)	0.650
Other complication	1 (0.9%)	2 (1.0%)	0.959
Clavien–Dindo I–II	6 (5.7%)	14 (7.0%)	0.644
Clavien–Dindo III–IV	5 (4.7%)	9 (4.5%)	0.938
30-day mortality	0 (0.0%)	1 (0.5%)	0.465
Length of hospital stay, median (IQR)	74 h (53–98)	72 h (47–97)	0.963
Uncomplicated AA	316 (35%)	577 (65%)	
Operation time, median (IQR)	33 min (24–47)	37 min (26–45)	0.213
Morbidity	9 (2.8%)	14 (2.4%)	0.704
Superficial SSI	1 (0.3%)	4 (0.7%)	0.471
Organ/space SSI	3 (0.9%)	5 (0.9%)	0.900
Ileus	2 (0.6%)	1 (0.2%)	0.256
Bleeding^a^	0 (0.0%)	2 (0.3%)	0.295
Pneumonia	1 (0.3%)	2 (0.3%)	0.941
Other complication	2 (0.6%)	0 (0.0%)	0.056
Clavien–Dindo I–II	5 (1.6%)	11 (1.9%)	0.727
Clavien–Dindo III–IV	4 (1.3%)	4 (0.7%)	0.385
30-day mortality	1 (0.3%)	0 (0.0%)	0.176
Length of hospital stay, median (IQR)	23 h (17–39)	35 h (23–43)	<0.001

AA: acute appendicitis; IQR: interquartile range; SSI: surgical site infection.

a Bleeding = intraoperative bleeding requiring blood transfusion.

When variables including time of day of appendectomy (daytime surgery and nighttime surgery), patients’ American Society of Anesthesiologists (ASA) physiological score (I–II and III or more), surgeons’ experience (resident and specialist surgeon), and complicated disease (complicated appendicitis and uncomplicated appendicitis) were inserted into multivariate analysis (binary logistic regression analysis, Enter-method), only complicated disease was associated with higher morbidity (odds ratio (OR): 4.52, 95% confidence interval (95% CI): 2.59–7.89, p < 0.001), as shown in Table 3.

**Table 3:** Predictors of higher morbidity after appendectomy.

Variable	Morbidity		
	OR	95% CI	p value
Complicated disease^a^	4.52	2.59–7.89	<0.001
Nighttime surgery	1.02	0.58–1.80	0.942
ASA class 3 or more	1.31	0.56–3.10	0.532
Resident surgeon	1.21	0.70–2.10	0.499
Age ⩾65 years	1.01	0.41–2.45	0.990
Sex, male	1.55	0.88–2.74	0.129

OR: odds ratio; CI: confidence interval; ASA: American Society of Anesthesiologists.

a Perforation with either peritonitis or abscess.

## Discussion

Although it is controversial whether appendectomy can be safely delayed (7, 11–13), it is often unnecessary to postpone operation as a shorter delay may increase patient comfort, enables quicker recovery, and decreases costs (4). In some studies, the risk of surgical site infections increased in correlation with postponing the operation (11, 14, 15). Consequently, here we study whether it is safe to perform appendectomy during night hours. According to this study, there was no difference in morbidity, mortality, risk of surgical site infections, and organ/space surgical site infections or in other complications regardless of time of day during the surgery. The length of hospital stay was shorter among those operated during night hours.

In this study, we report postoperative morbidity and mortality lower than reported previously in the literature (16–18). Of all patients, 4.8% suffered from postoperative complications and 0.2% died. None of the deaths were associated with acute appendicitis or surgery itself. There were no intraoperative complications. The results cannot be explained by a lower rate of complicated disease—one in four of the operated patients had complicated disease, which is more than reported previously in the literature (19). In general, the operative results of the hospital were better than average, which can be seen in the material. Complications reported in this study are similar to that reported in the literature before (20). The most common was organ/space surgical site infection, of which majority required percutaneous drainage later. The risk was not associated with time of day. Furthermore, patient, disease, and surgery-specific demographics were similar in both groups.

In contrast to previous studies, both morbidity and mortality rates were similar regardless of time of day (7). We emphasize that it is not always necessary to perform appendectomies immediately, and it is may be safer to delay at least difficult operations if immediate surgery is not required to working hours. However, we encourage that appendectomy can be performed safely regardless of time of day if doing so. Furthermore, appendectomy is a quick operation and in case of emergencies requiring immediate surgery, it should not cause delay to these operations. In this study, operation time was similar in both groups, while patients operated during night hours had a slightly lower length of hospital stay.

The impact of fatigue on surgeon’s surgical skills has been evaluated with the help of simulators, for example. Studies have shown a consistent decline in cognitive function, but in technical skills, the outcomes have been mixed (9). In some articles, it was shown that acute partial sleep deprivation can also have positive short-term effect on cognitive skills and therefore enhanced technical performance with increased objective alertness within complex tasks (21). Despite increase in workload and sleepiness, subjects were able to learn new things proficiently and complete the tasks given (22). Also, operating during nighttime does not necessarily cause excessive fatigue if sufficient recovery time before and after on-call shifts is provided. A wide systematic review (23) on resident’s duty hour restrictors in surgery also showed that restrictions were not associated with improvements in surgical resident’s wellbeing. The review also revealed that restrictions had some negative impacts on patient outcomes and performance on certification exams.

The study has some limitations. This is a retrospective single-center study. However, a high number of patients were included in this study. In addition, the data are comprehensive and postoperative outcomes are precisely recorded for all patients. These data were collected in a high-volume tertiary care center; therefore, it is uncertain whether the results can be applied to smaller hospitals, and to hospitals where appendectomies are usually performed by the residents in the beginning of their specialization. Whether the surgeons’ experience influences the postoperative outcomes requires more research.

We conclude that nighttime appendectomy is safe and not associated with increased risks. All outcome measures in this study, including morbidity, mortality, length of the surgery and amount of intraoperative bleeding were similar regardless of time of day of the surgery. Therefore, we recommend that appendectomy would be performed without unnecessary delay even during nighttime, if needed.
